# Imaging Insights Into Abdominal Wall Function

**DOI:** 10.3389/fsurg.2022.799277

**Published:** 2022-02-25

**Authors:** John W. Read, Nabeel Ibrahim, Anita S. W. Jacombs, Kristen E. Elstner, Jeni Saunders, Omar Rodriguez-Acevedo

**Affiliations:** ^1^Department of Clinical Medicine, Faculty of Medicine and Health Sciences, Macquarie University, Sydney, NSW, Australia; ^2^Macquarie Medical Imaging, Macquarie University Hospital, Sydney, NSW, Australia; ^3^Department of Surgery, Faculty of Medicine and Health Sciences, Macquarie University, Sydney, NSW, Australia; ^4^Hernia Institute Australia, Sydney, NSW, Australia; ^5^Department of Surgery, Macquarie University Hospital, Sydney, NSW, Australia; ^6^Faculty of Medicine and Health Sciences, Macquarie University, Sydney, NSW, Australia; ^7^School of Medicine, University of Notre Dame, Sydney, NSW, Australia; ^8^Spine & Sports Medicine, Sydney, NSW, Australia

**Keywords:** abdominal wall, functional anatomy, complex ventral hernia, Botulinum toxin A, computerised tomographic images

## Abstract

**Purpose:**

The successful repair of any complex ventral hernia requires a thorough understanding of the underlying anatomical defect and its functional context. We describe an improved “functional” approach to CT imaging of the abdominal wall that can facilitate this understanding and assist surgical planning.

**Methods:**

This invited article reports the observational experience gained from the functional abdominal wall CT examinations of 88 patients who underwent complex ventral hernia repair using pre-operative Botulinum toxin A (BTA) infiltration of the lateral oblique abdominal muscles as well as a further eight patients with diastasis rectus abdominis who were examined to exclude ventral hernia.

**Results:**

The use of a functional CT protocol which supplements resting images with additional “crunching” images (acquired with the abdominal wall muscles all strongly contracted) can significantly improve the demonstration of ventral hernia defects. Crunching acquisitions can also help differentiate true hernias from dysfunctional bulges, identify muscle denervation or atrophic changes, reveal otherwise occult hernias that may be missed on resting or Valsalva images alone, and assist the pre-operative assessment of BTA effect.

**Conclusion:**

A more functional approach to pre-operative CT imaging of the abdominal wall can significantly improve the understanding of complex ventral hernia defects and help formulate effective surgical plans that achieve low recurrence rates and good functional outcomes.

## Introduction

The abdominal wall functions to contain and compress viscera, move the trunk and assist in stabilization of the spine and pelvis. This complex arrangement of muscles, tendons and fascia integrates with the spine, diaphragm and pelvic floor to form a continuous functional envelope. In effect, the abdominal wall constitutes one component of a highly flexible yet enormously strong suspension system that can dynamically tension for rigidity while simultaneously applying specific differential vectors of force required for precise movement and control of the trunk.

For the reconstructive surgeon attempting an effective repair for complex ventral hernia, the preservation of normal abdominal wall function remains a severe challenge. Pre-operative Botulinum toxin A (BTA) infiltration of the lateral oblique muscles has recently been reported as a useful technique to facilitate the closure of large ventral hernia defects (1), and in many cases can help to preserve normal function by avoiding the permanent loss of fascial integrity that would otherwise accompany TAR. This technique is a *functional* method of “lengthening” the abdominal wall by simply relaxing or reducing muscle tone.

Cross-sectional imaging performed before BTA injection, after BTA injection, and following operative repair offers insight into the functional aspects of both normal abdominal wall anatomy and structural derangements. This article will discuss the experience of the authors in using functional computed tomography (CT) to assess a large series of complex ventral hernia repairs assisted using pre-operative BTA injection (2). All 88 complex ventral hernia patients in our series met one or more of the following criteria: (a) fascial defect size ≥5 cm as either a single defect or the summed total of multiple “Swiss-Cheese” defects; (b) a loss of abdominal domain ≥20%; or (c) a recurrent or multi-recurrent ventral hernia. There were 53 males and 35 females with a median age of 64 years 4 months (range 21–84 years) and mean BMI of 31.4 ± 6.6 kg/m^2^ (range 21.8–60.9 kg/ m^2^). CVH type was classified as midline incisional in 81, midline primary/umbilical in 5, and midline traumatic in 2. The mean width of hernia defect was 12.9 ± 5.2 cm (range 6–30 cm) and there were 22 patients with loss of abdominal domain ≥ 20%.

Further insight into abdominal wall dysfunction was gained from experience with an additional small series of eight patients with diastasis rectus abdominis who underwent functional CT examination in the clinical context of abdominal bulge and/or chronic recalcitrant lumbar pain to exclude a diagnosis of midline ventral hernia (unpublished). All 8 cases had a measured transverse inter-rectus distance of more than 2 cm on resting CT images (mean 3.5 cm, range 2.2–6.0 cm). There were seven females and one male with a median age of 49 years (range 37–70 years).

## Normal Function

**Figure 1:**
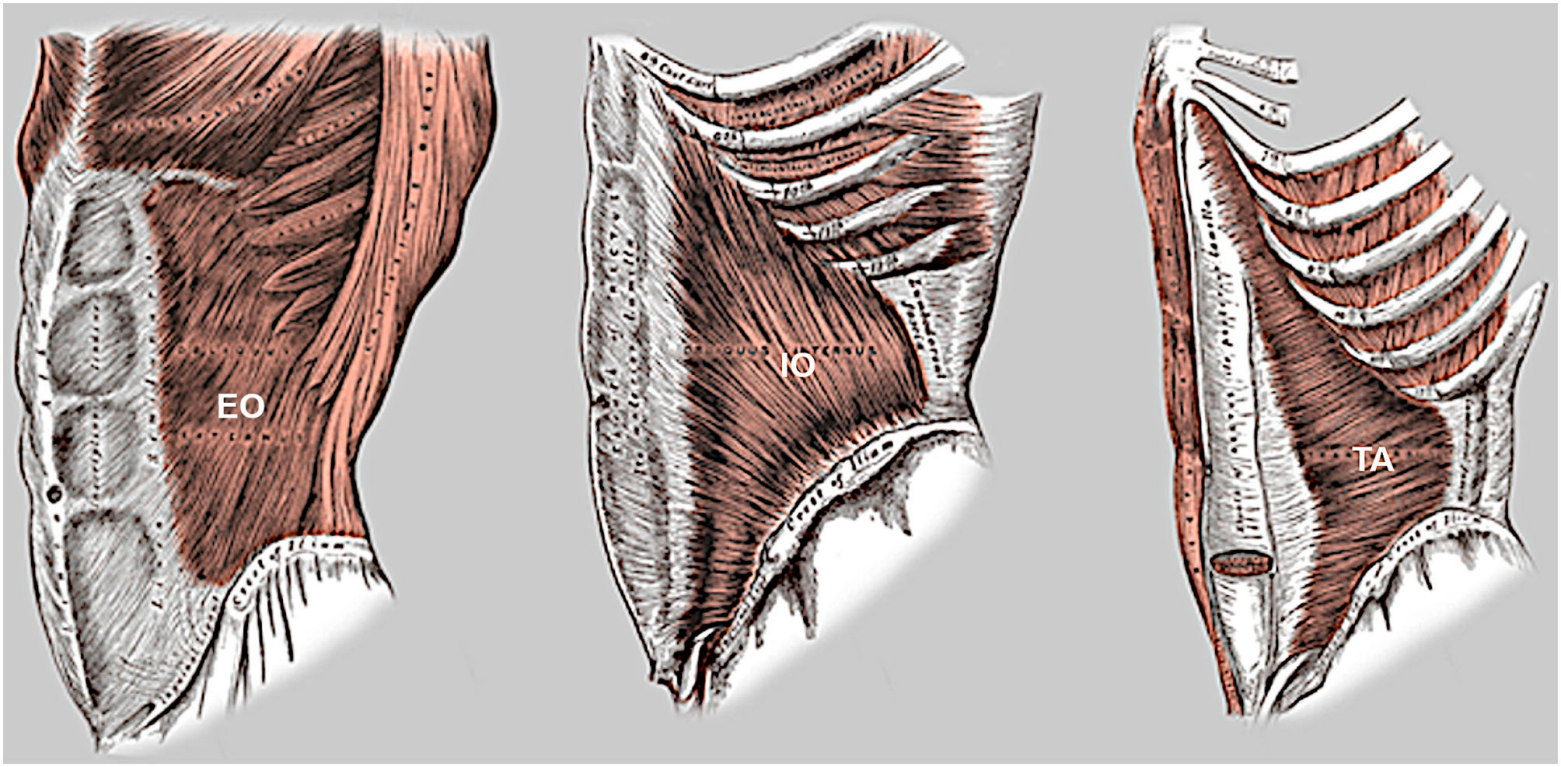
Lateral oblique muscles of the abdominal wall. Note the opposed fiber orientation of the external oblique (EO) and internal oblique (IO) muscles, the oblique inferomedial orientation of the lowermost fibers of both transversus abdominis (TA) and internal oblique below the level of ASIS, and extension of the upper segment of TA muscle into rectus sheath.

**Figure 2:**
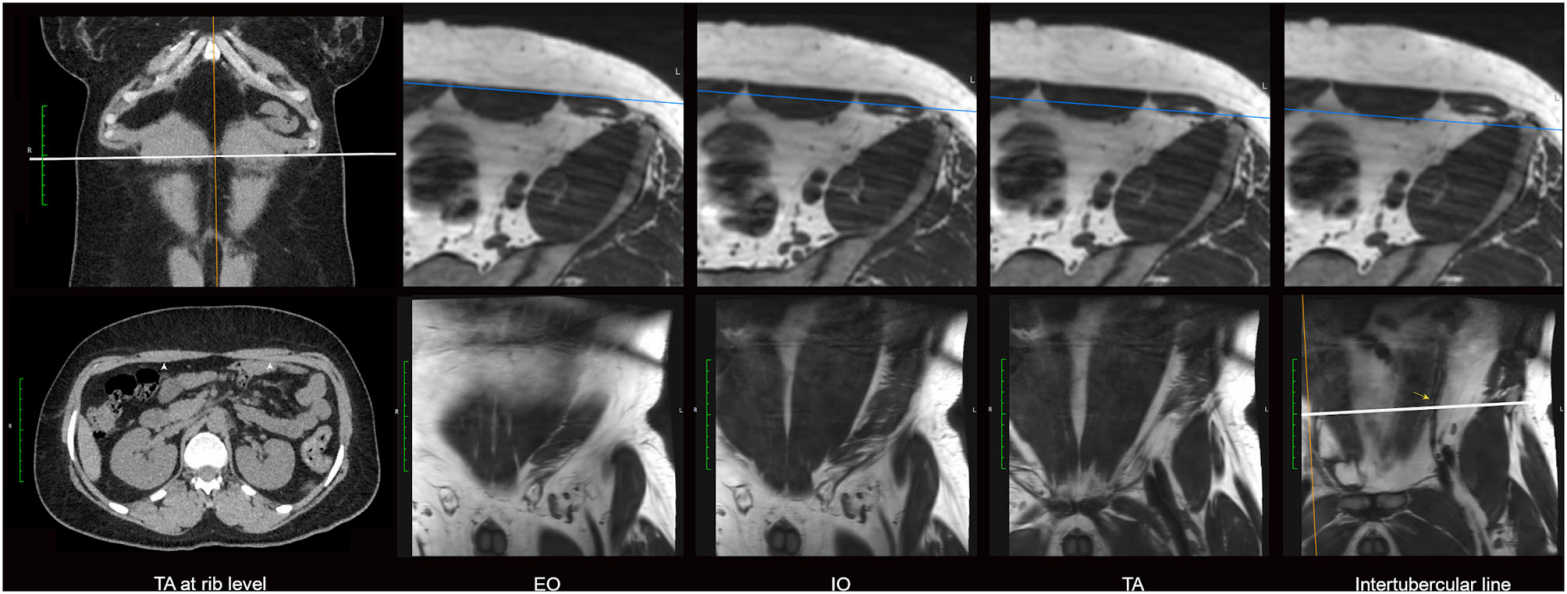
Anatomical arrangement of the upper & lower muscle segments of abdominal wall. CT images on the left show TA muscle (arrowheads) at rib level entering the rectus sheath posterior to each RA muscle. The remaining images are axial and sequential coronal oblique MRI sections obtained through the muscle layers of left lower quadrant abdominal wall in a male subject. Note the inferomedial long-axis fascicular orientation of all layers (EO, IO and TA) below the level of intertubercular line (indicated by white line on the far-right image of bottom row) toward the zone of pubic attachment and adductor cross-linkage. The arcuate line is not clearly resolved by imaging, but its approximate location can be suggested by the subtle angular change (yellow arrow) in the longitudinal course of the inferior epigastric vessels. The level of arcuate line varies between individuals but most commonly lies just above the intertubercular line.

**Figure 3:**
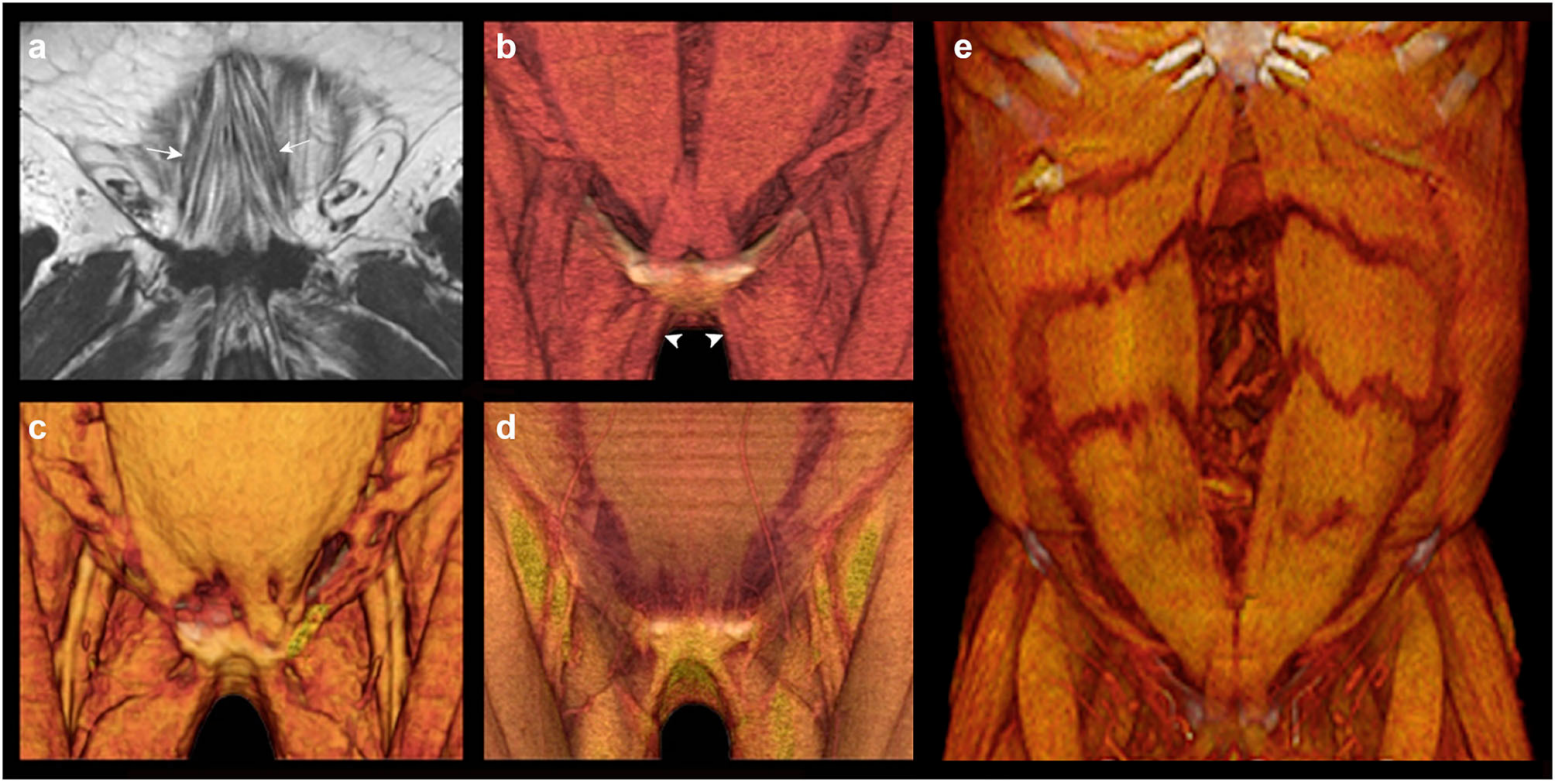
Pyramidalis anatomy. The pyramidalis muscles can be absent or unilateral in 20% of humans (3). **(a)** Coronal MRI of the pyramidalis muscles (arrowed) in a subject with interstitial fatty infiltration illustrates the typical oblique fascicular orientation; **(b)** 3D volume rendered CT of normal pyramidalis anatomy with muscles present bilaterally; note co-linear alignment of the pyramidalis muscles with the ipsilateral adductors of thigh (arrowheads); **(c)** 3D volume rendered CT shows unilateral absence of pyramidalis on the right; **(d)** 3D volume rendered CT shows bilaterally absent pyramidalis muscles with a consequent thin segment of only tendon & fascia visible in the immediate suprapubic zone; **(e)** 3D volume rendered CT in a case of extensive diastasis rectus abdominis shows the lower margin of divarication delimited by the upper margin of pyramidalis.

The abdominal wall is comprised of 5 paired muscles: External Oblique (EO), Internal Oblique (IO), Transversus Abdominis (TA), Rectus Abdominis (RA) and Pyramidalis (Figures 1–**3**).

The EO and IO muscles have an orthogonal oblique fascicular orientation that acts in various ipsilateral and contralateral combinations to produce trunk rotation and/or lateral spine flexion. At pubic level, there is an important functional cross-linkage between the fibers of (i) the EO and contra-lateral adductor longus muscles via the medial crus of superficial inguinal ring (4), and (ii) the IO and adductor muscles on both sides via a thick broad interconnecting pre-pubic plate of tissue described by anatomists as the anterior pubic ligament. This zone of pre-pubic confluence forms a critical hub of anterior force transmission between trunk and thigh during activities of trunk rotation and lateral spine flexion, as the contra/ipsilateral EO/IO and adductor muscles co-contract in appropriate combinations to facilitate the required motion.

The paired TA muscles have an opposed transverse fascicular orientation that laterally tensions the abdominal wall across the midline to facilitate spinal stability (5). These muscles should always synchronously co-contract and are the principal dynamic determinant of transverse abdominal wall contour. When activated they create a semi-rigid abdominal cylinder that integrates posteriorly with the fascia of multifidus to effectively brace the spine (6, 7). This stabilizing role of TA is supported by a predominant “slow twitch” fiber composition capable of sustained contraction, with a maximum contraction of only 5% required for the usual activities of daily living and 10% for rigorous activities (8, 9). Effective TA function requires an intact abdominal midline.

The paired RA muscles have a sagittal fascicular orientation which acts primarily to flex the trunk but can also assist trunk rotation and lateral bending. Transverse ‘tendinous intersections’ typically divide these muscles into at least 4 separate segments. The location of these intersections at umbilicus level and above corresponds with the lumbar vertebral column and suggests a probable “hinge” function during RA contraction that facilitates the concave abdominal wall contour required for trunk flexion. An intact and strongly contractile RA muscle is required to form a rigid column against which the lateral oblique muscles can effectively pull along their line of individual fascial attachment at linea semilunaris.

Although the structural anatomy of the rectus sheath has been well-described, its functional implications have not been given as much attention. The aponeuroses of the lateral oblique muscles above the level of arcuate line symmetrically envelop the RA muscles to ensure a strong anchor for optimal and effective control of the trunk above the level of sacrum. At rib cage level, the TA muscles extend medially *into* the rectus sheath behind the RA muscles (10) (Figure 2), presumably to improve mechanical purchase medial to the costal margin and assist the diaphragm in roles of both respiration (aiding expiratory power) and “core stability” (supporting the spine). The arcuate line is a key surgical landmark which constitutes the lower end of posterior rectus sheath. It is usually located just above the level of ASIS and results from a change in the arrangement of IO-TA decussation at the lateral margin of rectus sheath in which the fascial fibers above that passed posterior to RA muscle now alter course to instead pass anterior to RA muscle and fuse with anterior rectus sheath and more inferiorly with the conjoint tendon. This alteration in course reflects a change in abdominal wall function from control and stabilization of the lumbar spine *above* the intertubercular line to control and stabilization of the pelvis *below* the intertubercular line (Figure 2). Below the arcuate line there are no RA tendinous intersections and the force vector exerted by the lateral oblique muscles is directed inferomedially toward the pubis as required for synergistic functional cross-linkage with the adductors of thigh.

The pyramidalis muscles have an oblique fascicular orientation (Figure 3a) that has been described by anatomists as acting to “tension” the linea alba. However these muscles show clear co-linear alignment on 3D rendered CT images (Figure 3b), and direct cross-linkage on anatomical dissection (4, 11), with the ipsilateral adductors of thigh, which strongly suggests an important synergistic role in stabilization and control of the pelvis. An additional hypothesis, suggested by the CT observation that these muscles typically delimit the inferior reach of large RA muscle divarications (Figure 3e), might be dynamic reinforcement of the lower abdominal midline (which would otherwise consist of only a thin plate of fascia and tendon at suprapubic level, Figure 3d). The pyramidalis muscles are variable in size and presence (Figure 3) but can be surprisingly large and powerful structures in young adults.

**Figure 4:**
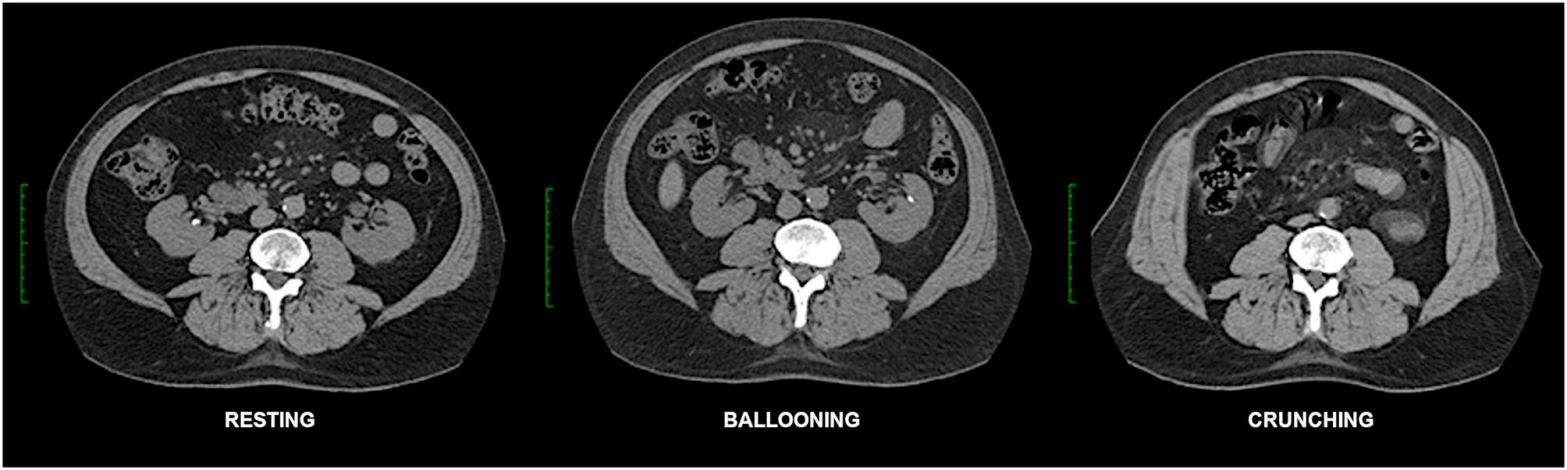
Functional CT of normal abdominal wall. Resting, ballooning and crunching phase axial non-contrast CT images obtained at the same vertebral level in this case illustrate normal abdominal wall function. Note preferential voluntary contraction of the *posterior* segments of the EO, IO and TA muscles on the ballooning images. Crunching images in this case show very strong uniform contraction of *all* abdominal muscle segments (as well as quadratus lumborum) on both sides. Normal fascial compliance is suggested by the absence of any transverse stretch of either linea alba or linea semilunaris on the straining images.

During upright locomotion and truncal movement all muscles of the abdominal wall function in an integrated fashion along with the pelvic floor and diaphragm to increase intra-abdominal pressure and further stabilize the lumbar spine. Optimal lumbo-pelvic and abdominal wall function requires global myofascial integrity and normal tissue compliance (Figures 4, **6**).

## Imaging Technique

CT is the cross-sectional imaging modality most often used to assess the abdominal wall. The advantages include a panoramic delineation of the pertinent anatomy, a fast scan time which largely eliminates movement artifact and allows the entire abdominal wall to be imaged in a single breath hold, and the acquisition of very thin section isotropic data which can then be reformatted in any plane without loss of resolution or be used for effective 3D rendering. Abdominal wall examinations *generally* do not require the use of either oral or IV contrast (12) but should ideally be performed as “functional” studies that include at least one straining image series in addition to the standard series acquired at rest. The disadvantages of CT include an exposure to ionizing radiation and a requirement for supine positioning on the scan table that is suboptimal for hernia demonstration.

Abdominal wall CT examinations are best undertaken with high performance low-dose multi-detector array scanners that employ exposure parameters and reconstruction algorithms appropriate for sub-millimeter resolution whilst keeping the X-ray dose within acceptable limits. As hernias can sometimes be difficult or impossible to appreciate without the use of provocative straining maneuvers (12) which increase intra-abdominal pressure, the authors routinely acquire scans of the abdominal wall in three separate “functional” phases (13): (i) at rest, (ii) while forcefully “ballooning” the abdominal wall outward as the spine remains flat against the scan table, and (iii) with the abdominal muscles all strongly contracted as the patient holds the shoulders and thorax about 5 cm off the scan table in an early sit-up (or “crunching”) position (Figure 4). An effective functional scan requires a compliant patient who can correctly and adequately perform these maneuvers.

**Figure 5:**
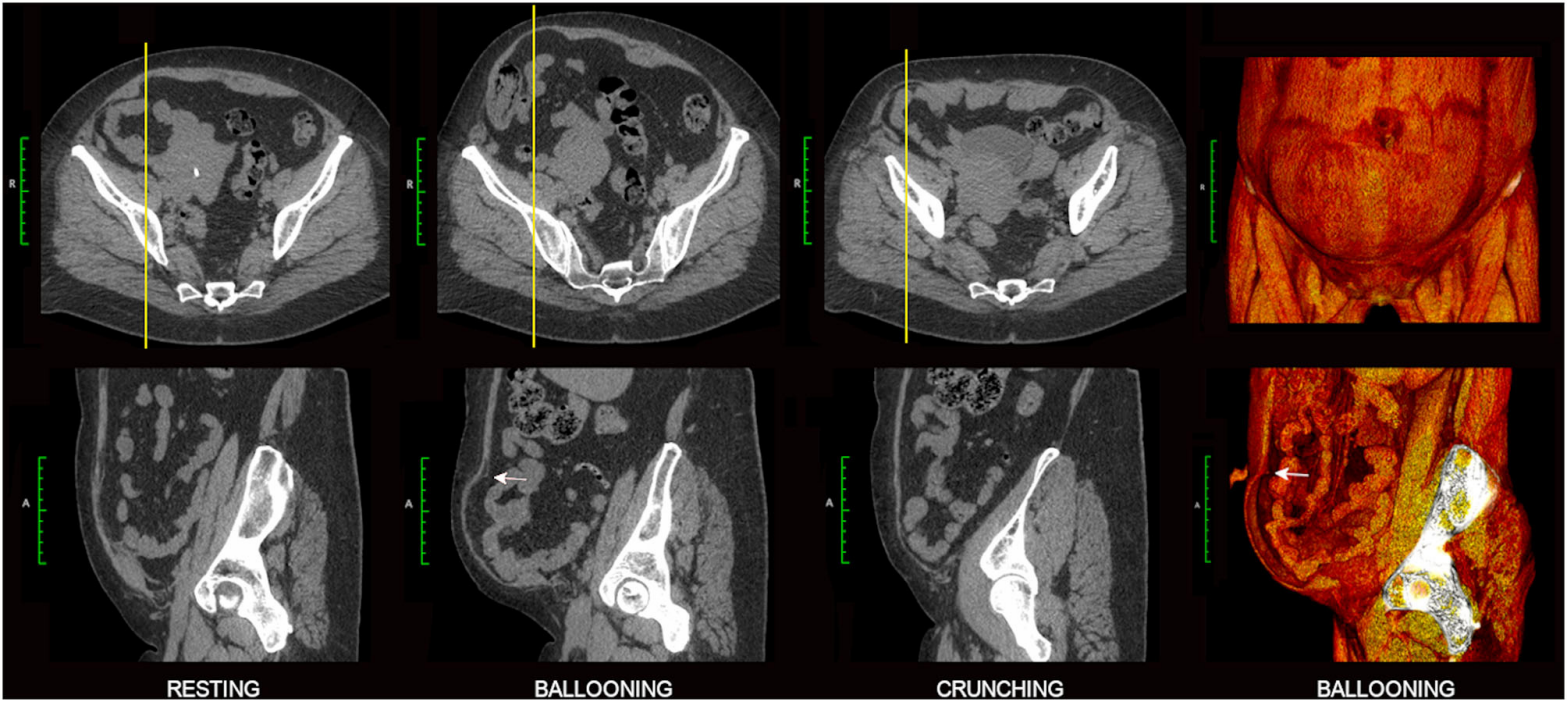
Value of ballooning CT acquisition. Axial and sagittal 2D images are shown from a 3-phase functional CT examination of a 50 year old female with broad-based right lower quadrant incisional hernia. Ballooning images best demonstrate the abnormality with characteristic abrupt “angular shouldering” (white arrows) at the margins of the hernia defect. Companion 3D volume rendered images on the right show the overall extent of lower quadrant bulge (frontal view, top) and profile the rim of the hernia defect (sagittal “cutaway” view, bottom).

Ballooning phase acquisitions [akin to a Valsalva maneuver (12)] *probably* raise the usual resting supine intra-abdominal pressure of ~6 mmHg to a level similar to that produced by standing (>20 mmHg) (14). An effective ballooning maneuver requires relaxation of both the RA muscles and *anterior* segments of lateral oblique muscles on both sides while simultaneously contracting the *posterior* segments of the lateral oblique muscles (Figure 4), incidentally illustrating the fact that the lateral obliques are under voluntary segmental control. In our experience, “ballooning” images are *only occasionally* superior to “crunching” images for the demonstration of hernias (Figure 5), and this acquisition can therefore be omitted from the *routine* scan protocol *if* there is concern for radiation dose as may be the case with younger patients. Nevertheless, in settings of high surgical risk such as the repair of massive ventral hernias, we believe the benefit of including a ballooning acquisition may outweigh any theoretical risk of increased radiation exposure.

**Figure 6:**
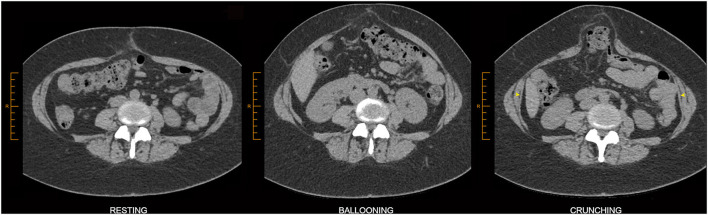
Value of crunching CT acquisition. This example of midline ventral hernia is best demonstrated on the crunching image. Crunching images are *either* equivalent *or* superior to ballooning images for the demonstration of ventral hernias in a great majority of cases. Associated TA muscle dysfunction can be appreciated here as relatively thin TA muscles on the resting image that show relatively poor contractility on the crunching image (yellow arrowheads). The straining images in this case also appear to show stretch of the linea semilunaris, a finding that has not yet been investigated for reliability but which might nevertheless raise *suspicion* of abnormal fascial compliance.

Abdominal “crunching” phase acquisitions aim for strong contraction of all abdominal wall muscles at the same time and are typically the most helpful functional acquisition. Crunching images generally provide the most sensitive and accurate imaging demonstration of abdominal hernias (Figure 6), presumably because they produce higher intra-abdominal pressures which more closely approximate those produced by other maneuver that involve stronger and more generalized muscle activation such as coughing or jumping (14). Crunching acquisitions also provide functional insight into muscle contractility that can be essential for distinguishing dysfunctional bulges from hernias, helpful in showing segments of muscle denervation or atrophy, useful in the assessment of pre-operative BTA effect, and valuable in discussing the likely prognosis of any attempted surgical repair.

**Figure 7:**
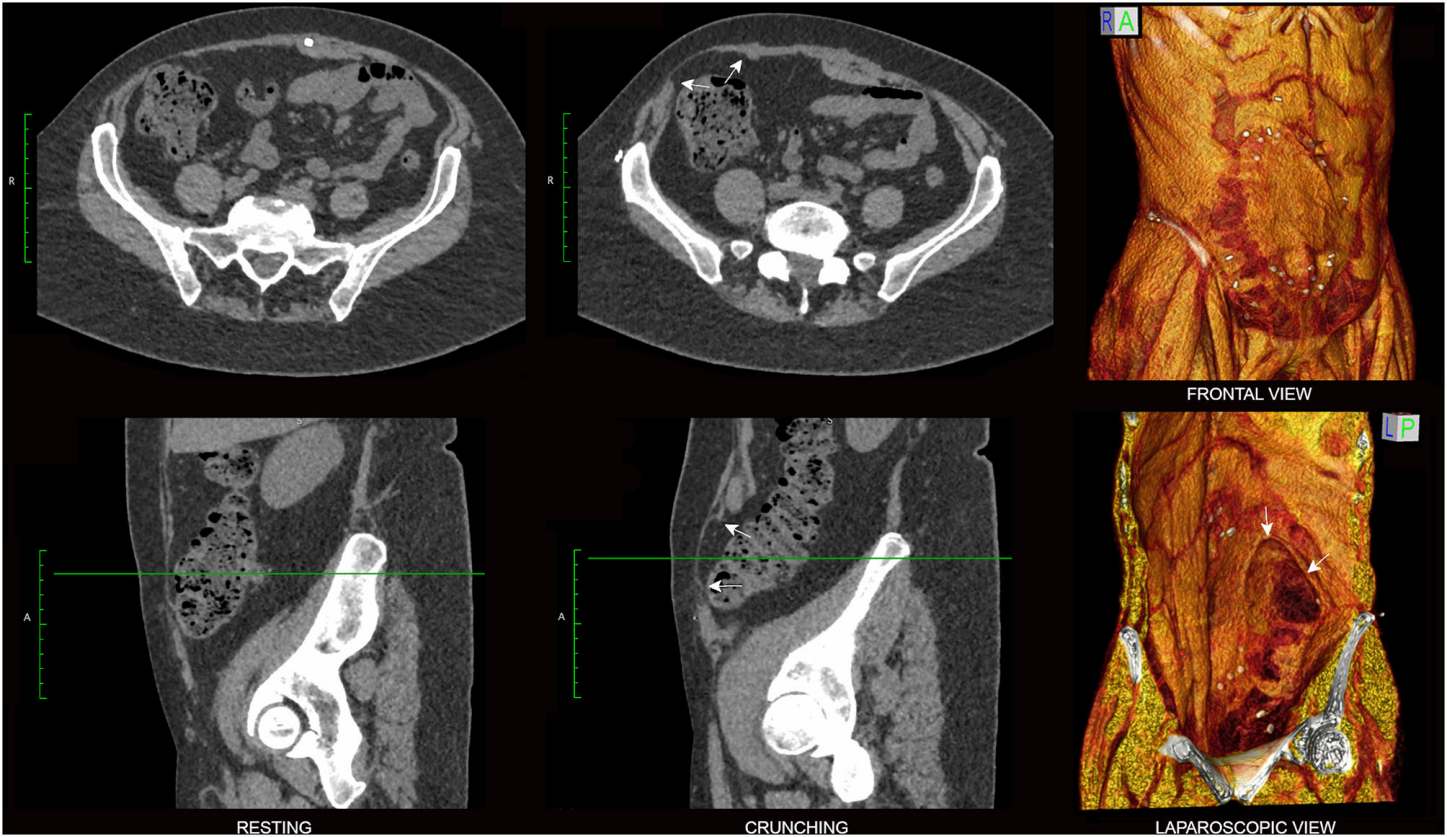
Hernia morphology. Resting **(left)** and crunching **(center)** phase axial **(top)** and sagittal **(bottom)** non-contrast CT images of a 58 year old female with right lower quadrant pain following TRAM flap procedure are shown with companion 3D volume rendered crunching images. In this case the differentiation of a dysfunctional bulge from shallow broad-based hernia was clinically uncertain, but the presence of abrupt ‘angular shouldering’ at the margins of the bulge (arrows) that was confidently visible only on the crunching phase CT images was regarded as diagnostic for hernia. Note mesh well-seen here as an ovoid non-anatomical “sheet-like” appearance overlying right RA muscle on the frontal view 3DVR image.

**Figure 8:**
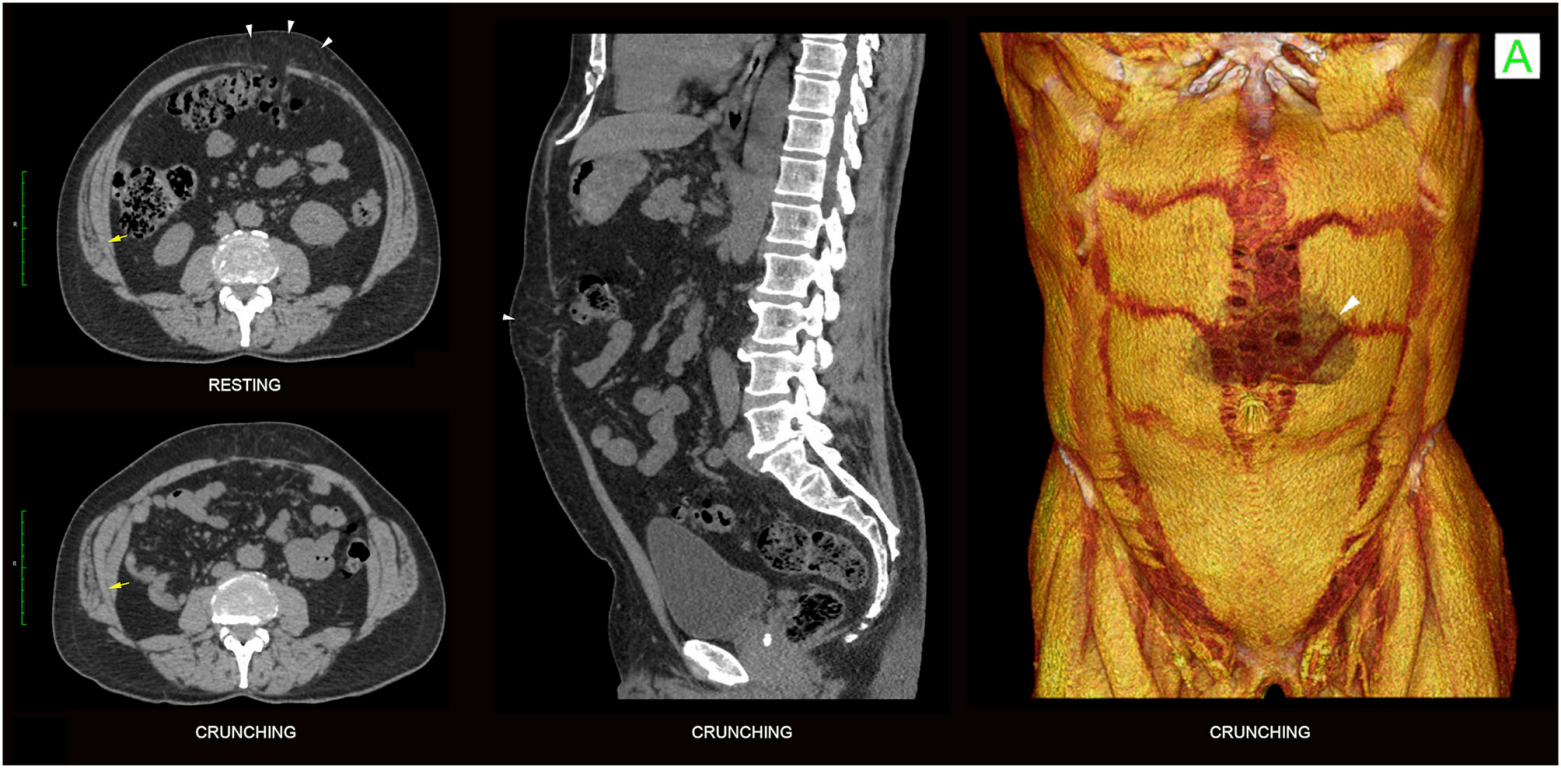
Value of supplementary 3DVR. Axial **(left)**, sagittal **(middle)** and 3D volume rendered **(right)** images are shown from the baseline functional CT examination of a 64 year old male with recurrent fatty ventral hernia (white arrowheads) on a background of previous colonic resection. 3D volume rendering provides immediate insight into the full extent of “swiss cheese” fascial attenuations and/or frank fascial defects that involve the diastasis segment of linea alba. An appreciation of the entire “hernia field” is one of the keys to planning an effective surgical repair with reduced risk of recurrence. Here, in this case of chronic midline compromise due to RA diastasis and superimposed ventral hernia, note strong overall compensatory EO + IO + TA muscle contractility (axial crunching images) that is sufficient to avoid any midline sagittal eventration bulge. Dysfunctional non-contraction of the *posterior segment* of right IO muscle is nevertheless appreciated (yellow arrows).

Image post-processing techniques such as 3D volume rendering (3DVR) or the real-time interrogation of anatomical detail using 2D multiplanar image reformations at a computer workstation can further assist the surgeon in complex cases (13) (Figures 5, 7, 8, **10**). Volume rendering can provide a more panoramic & holistic understanding of abdominal hernias (Figure 8), occasionally revealing additional unsuspected hernias that may be inconspicuous and easily overlooked on standard 2D images alone. They can also aid the perception of dysfunctional bulges/eventrations, muscle denervations and/or atrophic changes, mesh placements, other post-surgical changes such as scarring, and any relevant skeletal changes (13).

## Diagnosis

In addition to a CT protocol which incorporates straining maneuver to optimize the assessment of abdominal wall function, another key to the imaging differentiation of hernias from normal variants and dysfunctional bulges is the confident demonstration of pertinent anatomy including muscle, fascia, peritoneum and related fat spaces. This may be challenging, as fascia and peritoneum are relatively thin structures that can sometimes be difficult or impossible to clearly resolve, particularly when they are orientated oblique to the axial plane of primary image acquisition, are affected by excessive image noise, obscured by slice thickness artifact, or marginated on both sides by fat or soft tissue. Non-visualization of the peritoneum is a common source of interpretational error, as extra-peritoneal fatty protrusions (both hernias and normal variants) which lack a peritoneal sac may then be confused with intra-peritoneal herniations of fat. For these reasons, thin slice primary image acquisitions that have been obtained with low noise and which offer isotropic sub-millimeter resolution (enabling multiplanar interrogation) provide the greatest diagnostic confidence.

The clinical differentiation of dysfunctional bulges from true hernias can be difficult, but careful interrogation of the relevant morphology on crunching CT images is often helpful in making this distinction. As true hernia defects are actual structural discontinuities that reflect underlying tissue failure/disruption, they typically show an abrupt non-anatomical change in tissue contour at the point of disruption that is best described as “angular shouldering” (Figures 5, 7). Such contour changes may be subtle, especially in shallow broad-based hernias, but can nevertheless be appreciated if care is taken to critically evaluate the entire perimeter of the protrusion in an appropriate orthogonal plane.

**Figure 9:**
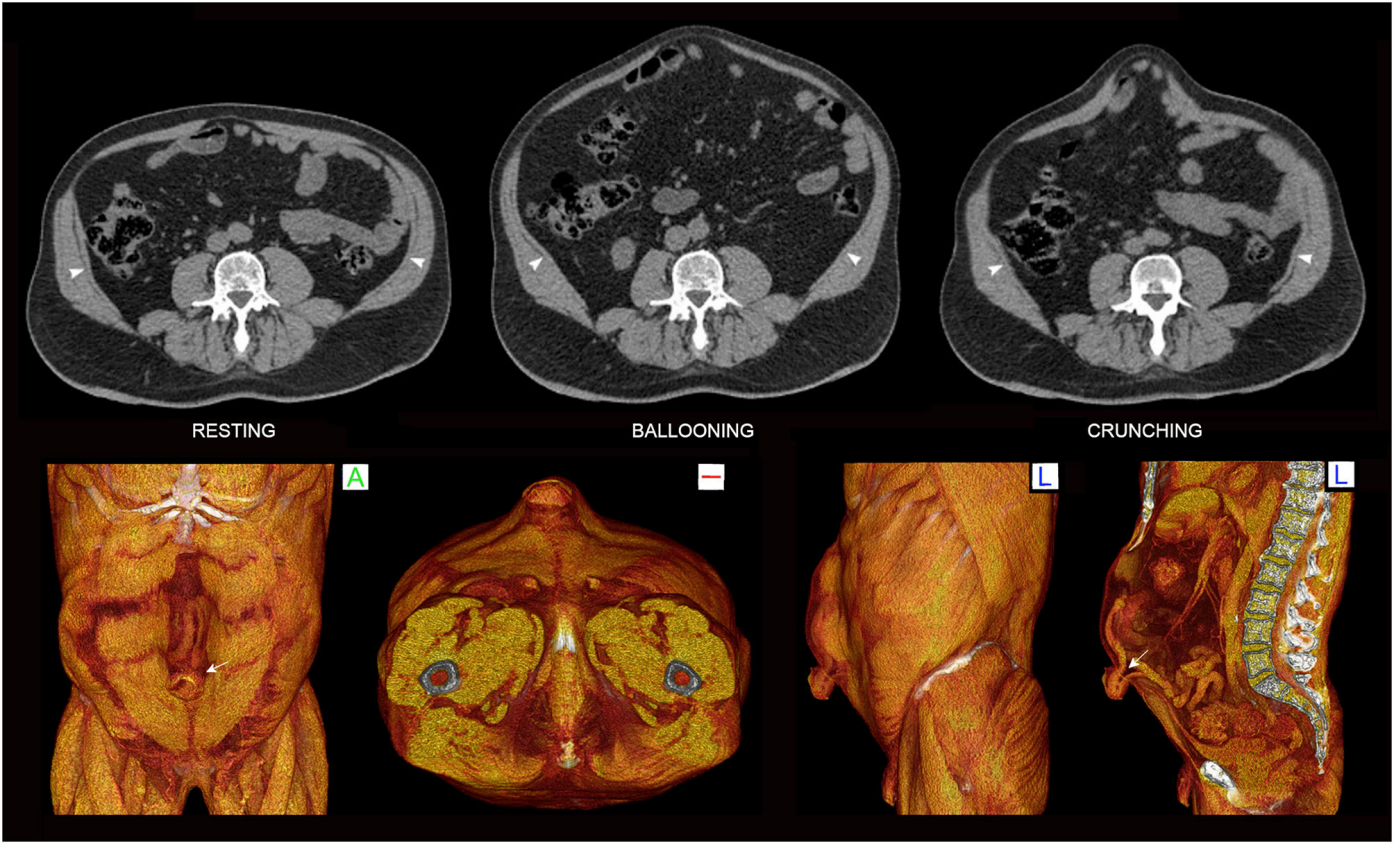
Diastasis rectus abdominis and superimposed umbilical hernia. Functional 3-phase 2D CT images obtained at supra-umbilical level **(top)** and crunching 3DVR images **(bottom)** show a small umbilical hernia with contained bowel (arrow) on a much larger background of diastasis rectus abdominis with associated prominent dysfunctional midline sagittal eventration bulge. Much of the bulge reflects poor TA function, appreciated as a lack of contractility on the ballooning and crunching phases (arrowheads) with associated eversion of the RA muscles along their medial edges. Note abrupt “angular shouldering” at the margins of the hernia (volume rendered images) vs. the continuous smooth contours at the margins of the dysfunctional bulge (2D images).

In contradistinction to hernias, cases of pure abdominal wall “dysfunction” have no underlying structural tissue failure or discontinuity (Figure 9) and therefore appear on crunching CT images as protrusions that show continuous smooth curved contours *without* abrupt angularity at the margins. Dysfunctional bulges are paradoxical “flail segments” that occur because the surrounding segments of abdominal wall contract and uniformly tension more effectively than the dysfunctional segment. They are commonly encountered in clinical practice and can have a variety of causes including muscle weakness (e.g. age, pain inhibition, deconditioning, denervation, dystonia), tissue elongation (e.g. diastasis rectus abdominis, surgical procedures such as TAR), and fascial hyper-elasticity [e.g. collagenopathies such as Ehlers-Danlos syndrome (15)]. It must be remembered that hernia defects and secondary dynamic dysfunctional bulges can, and often do, occur in combination (Figure 9).

**Figure 10:**
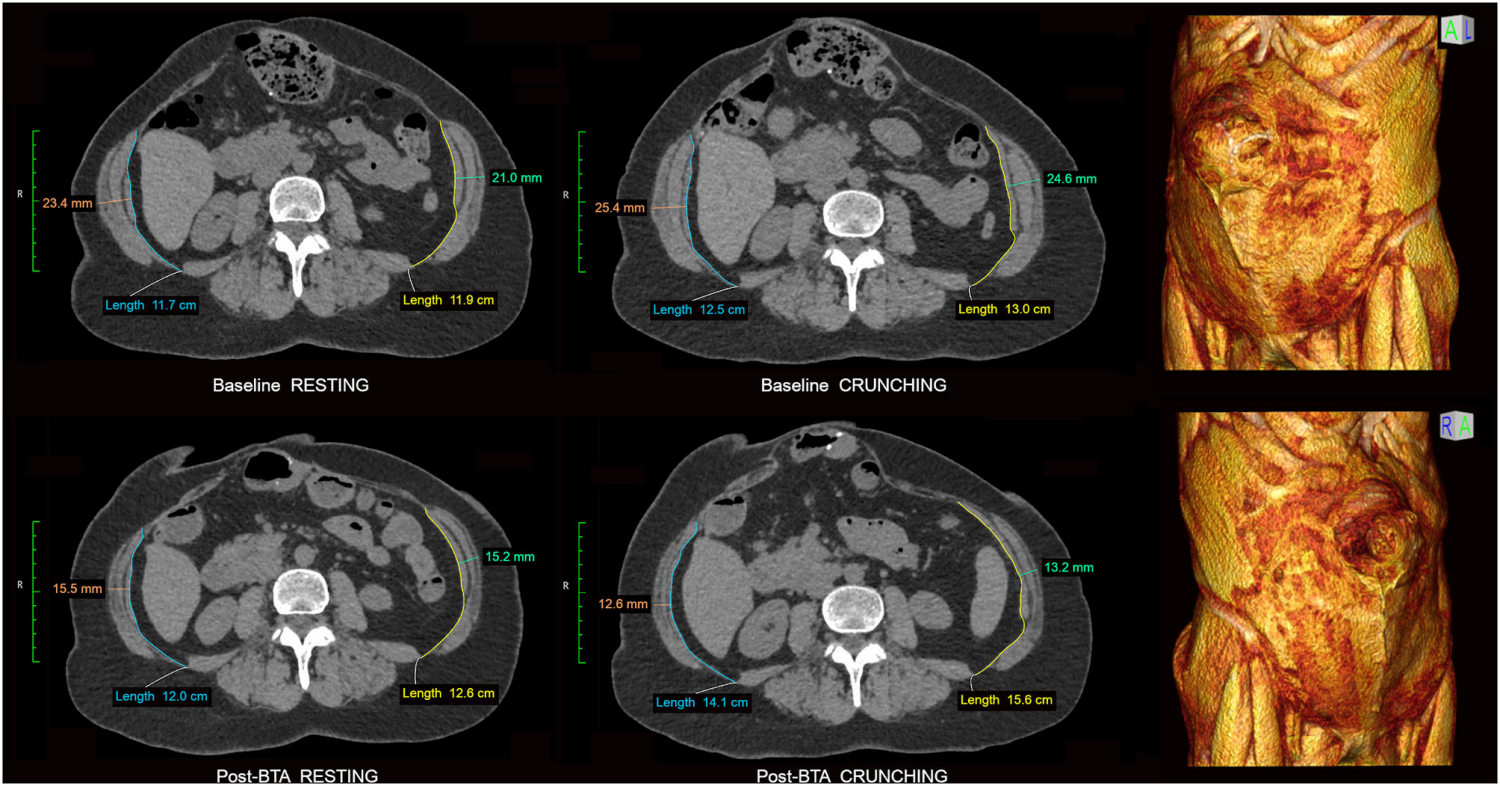
Measurement of BTA effect. Resting and crunching CT images obtained at the same supra-umbilical level are shown from the baseline and post-BTA pre-op examinations of a 67 year old female with recurrent midline incisional hernia. Companion baseline 3DVR images clearly show detachment of previously placed mesh along the upper and right margins of the recurrent hernia but importantly also demonstrate extensive bilateral RA muscle atrophy. Note very poor or absent contractility of both TA muscles on the baseline scan with relative IO muscle overactivity as a compensatory phenomenon. BTA effect can be measured as a percentage increase in axial length and/or reduction in overall thickness of each EO + IO + TA muscle complex following injection. In this case the right and left sides respectively show only a 1.6 cm (13%) and 2.6 cm (20%) increase in length but a 12.8 mm (50%) and 11.4 mm (46%) decrease in overall thickness on the crunching images. Several points are illustrated here: (i) Crunching images are generally better than resting images at gauging the degree of BTA effect; (ii) Muscle thinning is a better gauge of BTA effect than muscle lengthening; (iii) Poor RA muscle function limits the degree of intra-abdominal pressure and abdominal wall stretch that crunching maneuvers can generate and may consequently underestimate the amount of *length* gain that is actually available to the surgeon at operation.

Normal muscles at “rest” are never truly flaccid and instead possess a resting “tone” that reflects a small percentage of motor units always being active at any one time. Muscle *contraction* above this resting baseline is appreciated on straining images as muscle belly shortening (primarily in the nett direction of fascicular long axis) with an accompanying increase in overall muscle thickness, whereas muscle *flaccidity* (e.g. BTA effect) is conversely appreciated as muscle lengthening and thinning. In our experience, CT measurements of lateral oblique muscle *length gain* on axial images obtained following BTA injection tend to underestimate the amount of length gain that is actually available to the surgeon at operation. This most likely reflects some degree of limitation in effective patient compliance when attempting the CT crunching maneuver, most obvious in individuals with poor RA muscle quality and contraction strength (Figure 10). Instead, the *degree of muscle thinning* shown on straining CT images predicts the presence and degree of BTA effect with greater reliability.

**Figure 11:**
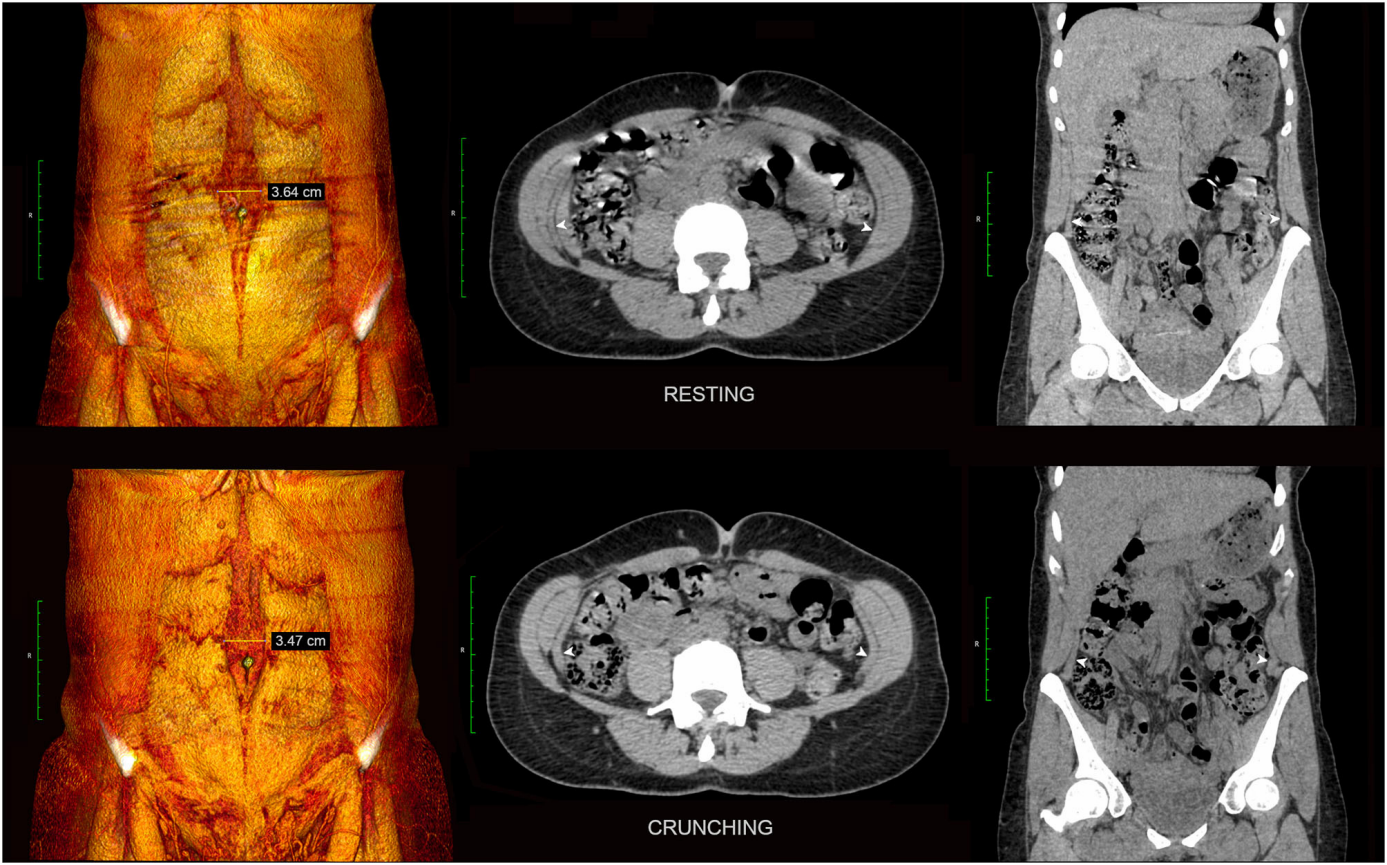
Diastasis rectus abdominis *without* dysfunctional bulge. Resting and crunching 3DVR **(left)** and axial/coronal 2D images **(center** and **right)** are shown from the functional CT examination of a 46 yearr old female with diastasis rectus abdominis. Note strong compensatory contractility of the entire EO + IO + TA muscle complex on each side (TA indicated by white arrowheads) sufficient in this case to maintain normal abdominal wall contour.

Both chronic abdominal wall dysfunctions and hernias/myofascial disruptions can produce varying patterns of compensatory muscle hypertrophy and/or “overactivity” (hypercontractility). Chronic TA muscle dysfunction is particularly common in patients with midline abdominal wall defects such as hernias and diastases. This can result in either (i) compensatory hypertrophy and/or overactivity of all muscles bilaterally (Figures 8, 11), or (ii) thin and poorly contractile TA muscles (Figures 6, 9) with compensatory hypertrophy and/or overactivity of the IO-EO muscles, noting that compensatory IO changes are often predominant (Figure 10). These changes are typically most pronounced at the level of the underlying midline abdominal wall defect and may be associated with a midline sagittal eventration bulge *if* the TA muscles are unable to generate sufficient transverse tension at the abdominal midline (Figure 9).

## BTA Effect

Pre-operative BTA infiltration of the lateral oblique muscles for patients undergoing complex ventral hernia repair has been shown to induce muscle flaccidity and elongation that assists defect closure (1, 2, 16, 17). We assessed the CT appearance of the individual lateral oblique muscles in a series of complex ventral hernia repairs before and after pre-operative BTA injection (18), with this review yielding some surprising observations.

**Figure 12:**
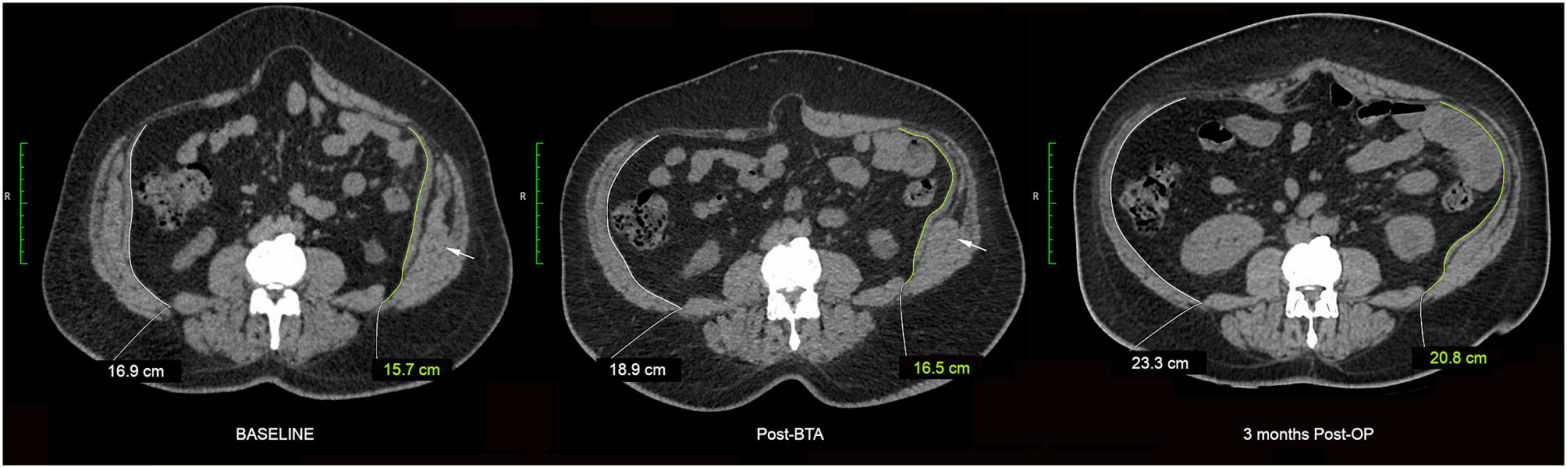
Variable BTA effect. Crunching phase images obtained at the same vertebral level are shown from the baseline diagnostic, pre-operative post-BTA and 3 months post-operative CT examinations of a patient with complex ventral hernia. Component relaxation was performed using a total of 300U BTA divided equally prior to the individual infiltration of each muscle layer of the abdominal wall in the anterior axillary line as described by Farooque et al. (1). The pre-operative post-BTA image shows variable chemical relaxation effect in the very same muscle: there is profound relaxation of the anterior segment but apparent non-relaxation of the posterior segment (white arrows) of left IO muscle. However the 3 months post-operative image subsequently shows (i) relaxation of the previously unresponsive posterior segment of left IO muscle, and (ii) an apparent increase in overall BTA effect as evidenced by a reduction in thickness and increase in axial length of the EO + IO + TA muscle complexes on both sides.

Observed contractility patterns in cases of both muscle dysfunction (Figure 8) and BTA-induced muscle relaxation (Figure 12) support the concept of EO, IO and TA muscle innervation as dividing broadly into anterior and posterior segments. In our group of patients with large longstanding hernia defects, the baseline diagnostic functional CT scans demonstrated a *segmental* pattern of impaired abdominal wall muscle contractility that was variable and unpredictable, with up to 14% of *either* anterior *or* posterior muscle segments in any layer found to be *non-contractile* prior to BTA injection (Figure 8). This observation would suggest that ventral hernia defects are a direct cause for segmental non-function of the lateral obliques, although we lacked any adequate control group to objectively confirm the hypothesis.

When the effect of BTA infiltration was assessed by individual layer on progress CT examinations obtained just prior to operation, we found that induced muscle flaccidity was unexpectedly absent in 30/132 (23%) segments, partial in 39/132 (29%) segments, and full in only 63/132 (48%) segments. Thus, despite an effective therapeutic response in terms of reduced overall EO + IO + TA thickness and increased EO + IO + TA length (Figures 10, 12) assisting defect closure at operation in all cases, just over half of all muscles injected showed a limited response to BTA just prior to surgery.

Progress *post-operative* scans performed 3–6 months *after surgical repair* also showed an unexpected *increase* in apparent muscle flaccidity in just over one-third of all segments (Figure 12). Interestingly, these progress scans also showed the “direction” of change in apparent BTA effect between the anterior and posterior segments of any given muscle layer to be concordant in only 28/66 (42%) and discordant in 26/66 (39%), with “discordance” deemed to be present if contractility was found to diminish in the anterior segment while at the same time appearing to increase in the posterior segment *of the same layer* (or vice versa). We therefore postulate that CT may underestimate the waning of BTA effect over time due to increased lateral traction forces acting on the EO, IO and TA muscles after surgical restoration of the abdominal midline (i.e. increased tension will tend to maintain a longer and thinner muscle despite any increase in resting muscle tone as BTA effect wanes). This phenomenon would not only account for an apparent increase in BTA effect over time but also the discordant direction of change in BTA effect between anterior and posterior segments of any given muscle layer as a consequence of the increased lateral traction force exerted by an increasingly active segment on a companion segment that remains flaccid.

Despite the demonstrated variability of BTA effect between individual muscle segments in our series, all cases achieved an overall effective therapeutic result at the time of operation. In actual clinical practice, we routinely compare baseline and post-BTA resting CT images to estimate the amount of lateral oblique length gain induced by BTA *prior* to operation (in our series averaging 4.1 cm per side) and use this information to decide whether additional techniques such as pre-operative progressive pneumoperitoneum or component separation will be required to achieve defect closure. It should be expected that BTA effect will be limited or absent wherever abdominal muscle is affected or replaced by a significant area of scar, atrophy, chronic denervation or mesh.

## Limitations

As this is an observational study that reports experience with a novel CT technique for assessing abdominal wall function in the sole context of complex ventral hernia, caution should be exercised in other clinical settings. The sensitivity and accuracy of this diagnostic technique for abdominal muscle function has not yet been validated against any independent gold standard. Crunching CT acquisitions require a subject who can understand and adequately comply with postural instructions to achieve an effective end-result. The value of functional CT as a test of fascial compliance has not yet been determined.

## Conclusion

An improved functional approach to CT image acquisition which includes a “crunching” acquisition can expand the diagnostic power and clinical utility of standard CT protocols that are the current mainstay of pre-operative ventral hernia imaging. A functional work-up can help the reconstructive surgeon better understand the extent of the “hernia field” and any associated dysfunction, better manage patient expectations, and plan an effective repair with reduced likelihood of subsequent recurrence. Functional CT examinations have provided new insight into abdominal wall function/dysfunction and the therapeutic effect of BTA. The overall degree and spatial distribution of BTA effect has shown unexpected variability while nevertheless achieving an effective therapeutic result at the time of operation.

## Data Availability Statement

The raw data supporting the conclusions of this article will be made available by the authors, without undue reservation.

## Ethics Statement

Ethical review and approval was not required for the study on human participants in accordance with the local legislation and institutional requirements. Written informed consent for participation was not required for this study in accordance with the national legislation and the institutional requirements. Written informed consent was not obtained from the individual(s) for the publication of any potentially identifiable images or data included in this article.

## Author Contributions

All authors listed have made a substantial, direct, and intellectual contribution to the work and approved it for publication.

## Conflict of Interest

The authors declare that the research was conducted in the absence of any commercial or financial relationships that could be construed as a potential conflict of interest.

## Publisher's Note

All claims expressed in this article are solely those of the authors and do not necessarily represent those of their affiliated organizations, or those of the publisher, the editors and the reviewers. Any product that may be evaluated in this article, or claim that may be made by its manufacturer, is not guaranteed or endorsed by the publisher.
